# The Impact of COVID-19 on the Support for the German AfD: Jumping the Populist Ship or Staying the Course?

**DOI:** 10.1007/s11615-022-00398-3

**Published:** 2022-05-02

**Authors:** Michael Bayerlein, Anne Metten

**Affiliations:** 1 Department of Political Science, Kiel University, Westring 400, 24118 Kiel, Germany; 2 Kiel Institute of the World Economy, Kiel, Germany

**Keywords:** Populism, COVID-19, Political parties, Voting behavior, Elections, Populismus, COVID-19, Politische Parteien, Abstimmungsverhalten, Wahlen

## Abstract

Populist parties enjoy stable support in various European countries. The literature on the rise of populism argues that this support especially increases in times of crises. Surprisingly, the German right-wing populist Alternative für Deutschland (AfD) did not increase its support in the wake of the COVID-19 pandemic. Moreover, the party even lost 2.3 percentage points in the 2021 federal election. We address this puzzle and ask why the AfD has not been able to use the crisis to its advantage. Our main argument in answering this question is that, although the AfD pursued the classic populist strategy of fundamental opposition, the support base of the AfD is strongly divided on the preference towards measures containing the spread of COVID-19. This division is reinforced by individual affectedness by the pandemic. Introducing a novel weekly dataset on voter preferences, we show that the AfD support base is strongly divided on the issue with approval of the government measures being a significant and substantial contributor to vote switching away from the AfD. Using regional-level data and a difference-in-differences approach, we further show that western German regions hit especially hard by the pandemic display a lower AfD vote share than other regions. Our findings have important implications for the impact of exogenous shocks on electoral competition and also on the future of populist parties.

## Abstract (de)

Populistische Parteien genießen in verschiedenen europäischen Ländern eine stabile Unterstützung. In der Literatur über den Aufstieg des Populismus wird argumentiert, dass diese Unterstützung vor allem in Krisenzeiten zunimmt. Überraschenderweise konnte die deutsche rechtspopulistische Alternative für Deutschland (AfD) ihre Unterstützungsbasis im Zuge der COVID-19-Pandemie nicht steigern. Darüber hinaus hat die Partei bei der Bundestagswahl 2021 sogar 2,3 Prozentpunkte verloren. Wir gehen diesem Befund nach und fragen, warum es der AfD nicht gelungen ist, die Krise zu ihrem Vorteil zu nutzen. Unser Hauptargument bei der Beantwortung dieser Frage ist, dass die AfD zwar die klassische populistische Strategie der Fundamentalopposition verfolgt, die Unterstützungsbasis der AfD aber stark gespalten ist, was die Präferenz für Maßnahmen zur Eindämmung der COVID-19-Ausbreitung angeht. Diese Spaltung wird durch die individuelle Betroffenheit von der Pandemie noch verstärkt. Durch die Nutzung eines neuen wöchentlichen Datensatzes zu den Wählerpräferenzen zeigen wir, dass die AfD-Unterstützungsbasis in dieser Frage stark gespalten ist, wobei die Zustimmung zu den Regierungsmaßnahmen einen signifikanten und wesentlichen Beitrag zur Abkehr von der AfD leistet. Unter Verwendung von Daten auf regionaler Ebene und einem Differenz-von-Differenzen-Ansatz zeigen wir außerdem, dass westdeutsche Regionen, die besonders stark von der Pandemie betroffen sind, einen geringeren AfD-Stimmenanteil aufweisen als andere Regionen. Unsere Ergebnisse haben wichtige Implikationen auf die Auswirkungen exogener Schocks auf den Wahlwettbewerb und auf die Zukunft populistischer Parteien.

## Introduction

The successes of populist parties is often attributed to economic, social, or political crises (Albertazzi and McDonnell 2008; Di Piramo 2009; Knight 1998). Based on this, it is reasonable to expect that the COVID-19 crisis should also have a positive effect on support for populist parties. Surprisingly, this does not seem to be true in every case, as the German right-wing populist *Alternative für Deutschland* (AfD) is losing, not gaining, both in polls and in the 2021 federal election. Focusing on the AfD and the 2021 federal election, our paper answers the question: Why is the COVID-19 crisis not having a positive effect on AfD support? We examine in detail the policy preferences of AfD voters, the attitudes exhibited by individuals who previously supported the AfD but now indicate a different voting intention, and how the AfD performed in regions hit especially hard by the pandemic.

While dealing with the pandemic is a challenge for all parties, the positioning and performance of the AfD is of particular interest. This is because populist opposition parties like the AfD are usually classified as ‘challenger’ parties, meaning that one of the core electoral strategies is to challenge the mainstream political consensus (see Hobolt and Tilley 2016). This challenge is characterized by a strategy of owning single issues related to the core ideology and generating support based on the parties’ positions on these issues (see Adams et al. 2006). This strategy is particularly successful in times of crisis because challenger parties can take controversial positions and propose solutions that generate attention and are located outside the political consensus (see Moffitt 2015). However, this strategy can only be successful if the position is shared by a certain part of the electorate (see Meguid 2005). Looking at the AfD’s strategic positioning, previous contributions show that the AfD attempted to use the COVID-19 crisis as a new opportunity structure to rally against the government, similar to the financial crisis and the increased influx of migrants 2015/2016 (Ruhose 2020; Wacker and Kieslich 2021).

In this paper, we argue that the COVID-19 crisis does not have a positive impact on AfD support because of an unprecedented internal division of the AfD’s support base on the issue. In comparison to other parties, this division is significantly larger and contributes to a loss of votes as previous voters of the AfD switch to other parties. We reason that this behavior is especially driven by affectedness by the pandemic and, therefore, more pronounced in regions that have been worse hit by the pandemic. The empirical analysis of these theoretical considerations is conducted using a novel panel dataset on preferences of AfD voters from ‘Corona Trendfrage’ surveys conducted by the Press and Information Office of the German government. The impact of the severity of the pandemic is assessed on the regional level by implementing a difference-in-differences approach with the severity of the pandemic modeled as an exogenous shock.

While other studies have already looked at the performance of populist parties in the pandemic (see e.g., Bobba and Hubé 2021; Wondreys and Mudde 2020), we identify a lack of research that looks in detail at voter preferences and the internal division of populist parties when it comes to cross-cutting issues outside their core ideology. We address this research gap by not only providing a theoretical answer to the lack of success of the AfD in the crisis, but also by embedding these considerations into a broader empirical analysis over the course of the pandemic with a rigorous identification strategy.

Our paper proceeds as follows: First, Sect. 2 reviews the relevant literature on electoral strategies of populist parties in terms of demand and supply with a specific focus on crises as well as the electoral consequences of the COVID-19 pandemic. Second, we introduce our theoretical arguments in Sect. 3 and derive testable propositions of our model. In the next Sect. 4, we outline the data and provide first descriptive insights. Section 5 presents the identification strategy and the estimation results. Finally, Sect. 6 concludes the paper and answers the research question.

## Demand and Supply of Populism in Times of Crises

To motivate our arguments, we draw on two strands of literature on populist parties: The literature on the demand and supply-side of populism in general and Germany in particular, especially in times of crises, as well as the literature on the COVID-19 pandemic and populist parties. The first strand usually focuses on economic and cultural determinants of populism with contributions showing how import shocks, labor market competition, and economic hardship (Autor et al. 2020; Burgoon et al. 2019; Colantone and Stanig 2018; Pástor and Veronesi 2021) as well as fear of migration, nativism, perceived loss of cultural hegemony, and status anxieties (Betz 2017; Gidron and Hall 2017; Margalit 2019; Riedel 2018), all usually connected to progressing globalization (see, e.g., Rodrik 2020), lead to an increased demand for radical policies previously not supplied by established parties, i.e., ‘the elite’.

Focusing on Germany, however, recent contributions have provided evidence that voters of the AfD are not systematically different when it comes to the individual socioeconomic predispositions (Lengfeld 2017). Rather, voters of the AfD are usually characterized by a demand for nativist and populist attitudes (Arzheimer and Berning 2019; Pesthy et al. 2021) and a grave distrust in political institutions as well as political dissatisfaction (Rösel and Samartzidis 2018; Schulte-Cloos and Leininger 2021). According to the literature, these attitudes as well as additional opportunity structures are especially pronounced in eastern Germany, where the AfD has had several strongholds at least since the federal election of 2017 (Diermeier 2020; Pesthy et al. 2021). Recent contributes argue that it is this demand for radical nationalist and nativist antimigration positions that the AfD has started to supply since its foundation in 2013, and which has been sharpened, especially in the wake of the influx of migrants and asylum seekers in 2015/16 (Arzheimer 2015; Bieber et al. 2018; Hambauer and Mays 2018). Based on this, Arzheimer and Berning (2019) conclude that the demand and supply-structures of the AfD now resemble those of other European radical right-wing parties with a strong focus on issues related to the GAL-TAN dimension of political contestation, especially migration and nativism (see also Bayerlein 2021).

The research specifically concerned with crisis and populism assumes a connection between various forms of economic, political or cultural crises and the electoral success of populist actors. The rationale behind this is that crises provide opportunity structures for the rise and success of populists (see, among others, Kriesi 2015; Moffitt 2015; Laclau 2005; Ruhose 2020). This is the case, as ‘challenger’, populist opposition parties such as the AfD pose a particular challenge to the political (mainstream) consensus (Hobolt and Tilley 2016) by focusing on and occupying individual issues (Adams et al. 2006). This strategy is particularly successful in times of crises, as challenger parties can take controversial positions and propose solutions that not only generate attention but are also located outside the political consensus (Moffitt 2015). This can break up the (mainstream) discourse and open up space for a (populist) counterdiscourse (Stavrakakis 2005). The existence of a crisis is thus treated as a condition through which populist parties emerge or persist. Focusing on the AfD, contributions have shown how the AfD used the ‘cultural’ and political crisis in the wake of the influx of migrants and asylum seekers as an opportunity structure to gain voters on its main dimension of political contestation (Geiges 2018; Stecker and Debus 2019; Wurthmann et al. 2021).

The second strand of research that we base this paper on focuses particularly on populism and the COVID-19 crisis and argues that the COVID-19 crisis differs from economic or migration crises in that it does not fit into the common problem-solving schemes of the latter crisis, through which populist parties have grown and/or played an active role (Bobba and Hubé 2021; Ruhose 2020). An exogenous crisis, i.e., one that is not determined by factors within the political system (such as natural disasters), is harder to politicize “since causal attribution of responsibility is not always possible” (Bobba and Hubé 2021, 7). What populist parties then try to do is to move the problem into the realm of human intention in order to follow the populist electoral strategy and blame ‘the elite’ for the crisis (ibid.). Populist parties have attempted to do so by emphasizing the Chinese origin (Trump’s ‘Chinese virus’), linking the pandemic to (‘illegal’) migration and/or ethnic minorities, or simply denying or downplaying the danger of the virus (Wondreys and Mudde 2020).

Similar to other crises, the ‘rally ’round the flag’ effect (see Mueller 1970), through which parliamentary opposition slips into the background, was initially observed in several countries (Kritzinger et al. 2021; Yam et al. 2020). However, as the pandemic progressed, the same populist parties that criticized the measures as too late or too little at the onset of the pandemic began to speak out against the alleged ‘antidemocratic’ and ‘unconstitutional’ nature of the government’s actions only a little while later (Wondreys and Mudde 2020). A similar path was also pursued by the AfD. After initially struggling to find a position that was also in congruence with the preference of its voters the party quickly returned to fundamental opposition (Ruhose 2020). Generally, populist parties have reacted ambivalently to the pandemic, which they have not been able to exploit for their own benefit in the initial phase, e.g., in the form of greater support (see Bobba and Hubé 2021; Wondreys and Mudde 2020).^1^

What has not yet been studied, however, is why populist parties, like the AfD, did not succeed in gaining ground. The vote loss of parties and especially the AfD in the 2021 election is, of course, not a monocausal relationship. Rather, research argues that the AfD has gone through rather tumultuous years since the 2017 federal election, with financial scandals and intraparty disputes shaking the party internally (Heinze and Weisskircher 2021; Jäger 2021), while at the same time being externally excluded from political participation in the German federal parliament and the state parliaments (Heinze 2021; Schroeder et al. 2020).

However, there has been no research to date on how the COVID-19 pandemic has contributed to the electoral losses of the AfD. It is necessary to examine in detail whether the preferences of the electorate and, in particular, the attitudes of the AfD’s (previous) supporters towards the government’s COVID-19 measures have contributed to the electoral losses of the AfD or whether the federal election outcome is just a result of the parties stagnating vote share in general. We provide evidence for this effect by outlining a theoretical mechanism and running regression analyses on why the AfD has lost supporters over the course of the pandemic as well as by running difference-in-differences regressions to control other sources of the loss of votes.

## The Impact of the COVID-19 Pandemic on AfD Support

**Fig. 1:**
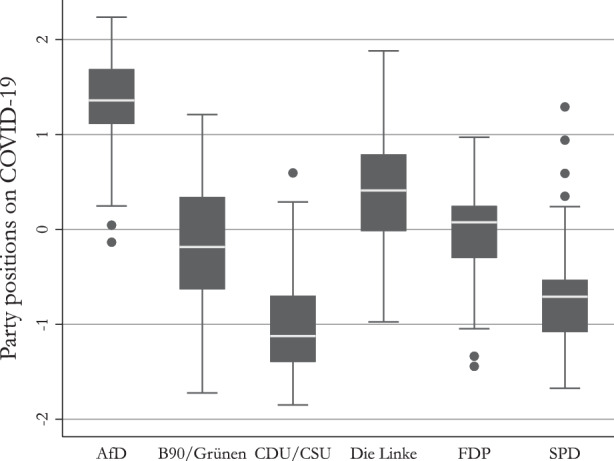
Average party positions on COVID-19 measures

For our theoretical argument, we focus on the demand side of electoral competition and the preferences of voters. Before addressing the demand side in greater detail, we briefly have to address the supply side of populism, i.e., the position the AfD took over the course of the pandemic, as the demand and supply sides are strongly interlinked and must be examined reciprocally. To shed some light on the policy position of the AfD towards COVID-19, Fig. 1 plots the policy positions of German parties on the COVID-19 measures taken by the government. The positions are calculated with the *Wordfish* tool introduced by Slapin and Proksch (2008) based on 93 parliamentary protocols between March 2020 and September 2021.^2^ Figure 1 shows that the AfD takes a distinct position outside the political consensus with high values indicating a position against the measures and low values indicating support of the measures.

While this strategy of fundamental opposition played out well for the AfD in the past in the wake of the migrant influx of 2015/2016 and the EU financial crisis, we argue that the COVID-19 pandemic does not create a similar opportunity structure to, for example, migration issues (see Arzheimer and Berning 2019). An opportunity structure that can be exploited by a populist party can only arise if two criteria are met. First, the issue has to be salient in the political discourse (see, e.g., Dennison 2020). Second, there needs to be a gap in the political supply structure that is matched by a political demand on the side of the electorate (see, e.g., Guiso et al. 2017; van Kessel 2013).

The salience of the COVID-19 pandemic was and is undoubtedly high for voters in different countries (see, e.g., Shino and Smith 2021). As was outlined above, the AfD took a position outside the political consensus of other parties and thereby fills a gap in the political supply structure. The question that arises is now, whether this supply is also matched with a demand on the side of the electorate. While the AfD’s electorate has a clear preference with regard to issues related to the cultural GAL-TAN policy dimension and, in particular, the rejection of globalization (Betz and Habersack 2019; Martin 2019; Rodrik 2020), it is unclear whether the same voters also have a demand for the COVID-19 policy position supplied by the AfD. This is especially the case, as the COVID-19 pandemic is a cross-cutting issue, which cannot be placed within the existing structures of electoral competition, i.e., the classic socioeconomic left-right and the cultural GAL-TAN dimension.

Having a strong and coherent support base with regard to the rejection of globalization, right-wing populist parties attempted to align their policy position taken towards COVID-19 with their position towards globalization by focusing on supply chains or the influence of migrants in the spread of the virus (Wondreys and Mudde 2020). Apart from these attempts, the COVID-19 crisis is an issue sui generis that does not easily relate to other policy issues and also distracts voters from these other issues (Bieber 2020; Wondreys and Mudde 2020). Hence, while a radical antiglobalization, antimigration, and pronationalism position in the GAL-TAN dimension has provided considerable support for the AfD, opposition to action on the COVID-19 pandemic does not fall into the same category, as, for example, strong and negative migration attitudes are something entirely different than preferences for wearing masks, social distancing, and compulsory vaccination.

Nonetheless, there are two possible connections between radical right-wing populist support and the rejection of COVID-19 measures. First, there is a strong historical connection between the (fascist) radical right-wing ideology and public health, as this ideology carries the notion of an imagined healthy ‘national body’ in which people with illnesses and disabilities are excluded from the imagined ‘people’ (Schäfer 2005). Solidarity with these marginalized and vulnerable minority groups in society is usually also rejected by right-wing populist actors, who rally against universal health care (Ammar 2018; Greer 2017; Speed and Mannion 2020) and often reject regulations on health risks like smoking (Falkenbach and Heiss 2021; Greer 2017) with a focus on promoting responsibility of the individual for remaining healthy. Although the AfD lacks a clear health policy (Wacker and Kieslich 2021), the notion of individualism and the rejection of the need to protect vulnerable individuals has been a central issue in the AfD’s stance on the COVID-19 pandemic since late March 2020 (Lembcke 2021).

The second commonality between AfD support and rejection of policy measures taken by the government against the COVID-19 pandemic is the fundamental opposition towards government policies, which is rooted in the political distrust as well as disaffection of voters, and captured by the anti-elite rhetoric of the AfD (Schulte-Cloos and Leininger 2021). Further, distrust and political disenchantment is also often rooted in individual or collective autocratic experiences (Xu and Jin 2018), arguably leading to greater resistance towards government limitations of one’s own civil liberties. This is especially in line with the finding that citizens in eastern Germany – against the backdrop of the end of the autocratic German Democratic Republic in 1990 – display higher distrust (Weisskircher 2020).

Based on the connection between right-wing populist support, attitudes towards health care and distrust in political elites, we expect a considerable amount of AfD voters to also reject the measures against the COVID-19 pandemic. However, the AfD is not only supported by politically entirely disaffected voters with deeply radical right-wing ideologies. Especially, these voters are likely to defect from the AfD if they disagree with the AfD’s policy position that does not easily align with the AfD’s position on the GAL-TAN dimension. As outlined above, we argue that the COVID-19 is a cross-cutting issue that is not fundamentally rooted in GAL-TAN issue dimension for which right-wing populist parties are known. Therefore, we expect a considerable number of supporters to also disagree with the AfD’s policy position and support the government’s measures against the COVID-19 pandemic. We, therefore, hypothesize:

### Hypothesis 1 

*The voters of the AfD are more divided on the issue of how to counter the pandemic than the voters of other parties.*

Now the question arises as to how AfD supporters with diverging political preferences behave. Based on the literature on voter–party congruence, we expect voters to select parties that are closest to them in terms of policy positions (see, e.g., Giger and Lefkofridi 2014). The spatial model of Downs (1957) suggests in this context that parties compete on an issue dimension and that voters will vote for a party that is closest to them on a particular issue or set of issues most important to them. Moreover, party–voter congruence, i.e., the closeness between party position and voter preference, is the greatest for issues that are particularly salient during the election campaign (Costello et al. 2021). This effect is driven by the fact that voters need to know which positions parties take on given issues. Voters can then select parties based on their current preferences, leading to high voter–party issue congruence (Andersen et al. 2005). This congruence is especially high for smaller parties formed around a dense ideological core, like challenger and niche parties, and in that sense also populist parties, as they focus on a small number of salient GAL-TAN topics, and their identification and reputation with these topics is all the stronger (Adams et al. 2006; Backlund and Jungar 2019; Costello et al. 2021).

Based on these considerations, we assume that voters do, indeed, vote for right-wing populist parties, like the AfD, based on issue proximity and accordingly do not vote for these parties if they are uncertain about the party’s policy position or if the party’s position is too far away from their own preference. Further, and based on our previous arguments, we conclude that new issues are more difficult to address, especially if they are cross-cutting issues and do not fit into a party’s broader portfolio.

Looking at the most salient issue of 2020 and 2021, the management of the COVID-19 pandemic and the measures to contain it have arguably been extremely salient during the German electoral campaign, in addition to other also relevant issues like Merkel’s succession. For the AfD, this high salience of COVID-19 means that the probability of losing votes increases if AfD supporters do not agree with the party’s position on how to handle the COVID-19 pandemic. As argued above, we reason that a considerable number of voters will not be in line with the AfD’s position of fundamental opposition to any measures against COVID-19 and the approach of radical individualism. While some voters still motivated by the now less salient nativist, antimigration position and their distrust in political elites might nonetheless keep supporting the AfD’s course of fundamental opposition, a considerable number will also jump ship and withdraw their support for the AfD by voting in accordance with their political preferences. From this, we derive the following hypothesis:

### Hypothesis 2 

*The stronger the support for measures to contain the COVID-19 pandemic, the higher the probability of withdrawing support from the AfD.*

Taking the argument on why supporters are turning away from the AfD one more step, we include additional considerations on individual affectedness, i.e., experienced severity of the pandemic. Based on our previous arguments, we reason that the effect of individual affectedness will be threefold. First, being confronted with a high number of cases in regional proximity and witnessing how people become ill or even die will likely have a positive impact on the individual support for measures to contain the spread of the virus. Second, the salience of the COVID-19 pandemic is likely to be even higher in regions that experienced a higher number of cases. Third, and going beyond our previous arguments, we argue that voters who are particularly affected by the pandemic also show more support for the government in the face of the existential threat posed by the COVID-19 pandemic.

In order to link the regional severity of the pandemic to loss of votes for the AfD and the support of government measures, we, therefore, take another look at the aforementioned ‘rally ’round the flag’ effect and the nature of the COVID-19 crisis. According to Mueller (1970, 21), the event that triggers the rally ’round the flag must be, among other things, “international [,] […] specific, dramatic, and sharply focused.” In his list of possible events to empirically prove the effect, military, economic, or political crises are the main triggers. A pandemic event that threatens not only many people’s health but also their very existence is a phenomenon that, in contrast to Mueller’s list, lies outside the sphere of political influence. In the same way as the crisis effects the supply side of populist politics, in that the government cannot be blamed for the emergence of the crisis (Bobba and Hubé 2021), the exogenous and live threatening nature of crisis is also likely to affect the demand side.

Based on this, we argue that the impact of this particular crisis has been so threatening that it has triggered existential fear. This existential fear has led many people to support the government’s course and its extensive measures to contain the pandemic as a precaution. We, therefore, argue that existential fear on the one hand increases the likelihood of people to support the government’s measures to contain the COVID-19 pandemic and withdraw their support from AfD. On the other hand, this existential fear also triggers a ‘rally ’round the flag’ effect and breaks the ’populist seduction’.

However, we assume that the described effect varies between and also within countries, as research concerned with crises and exogenous shocks has shown that individual proximity to an event is a major factor in moderating an individual perception of and affectedness by an event (Andersson and Bateman 2000; Loewenstein et al. 2001; Lujala et al. 2015; Weber 2013). This also applies to the pandemic, as the spread of the virus shows considerable between- and within-country variance (Allel et al. 2020; Gollwitzer et al. 2020; Sorci et al. 2020), introducing various levels of relative individual affectedness (see, especially, Li et al. 2021). Hence, we expect the described effect to be especially pronounced in areas where the incidence of infection, i.e., the threat level, is higher.

The regional severity of the pandemic is, therefore, possibly a crucial component in explaining the loss of votes of the AfD, as this affectedness likely (1) increases the preference for the implementation of measures to counter the pandemic, (2) raises the salience of the COVID-19 pandemic in the run up to the election even further, and (3) favors the development of a rally ’round the flag effect that draws away support from any opposition party towards the governing parties and their policy response. Based on this, we expect more individuals to switch from the AfD to another party in the regions especially affected by the pandemic, i.e., counties that experienced a more severe pandemic. We test this argument with the following hypothesis:

### Hypothesis 3 

*The worse a county is hit by the COVID-19 pandemic, the higher the loss of votes for the AfD* *.*

In conclusion, we make three arguments with respect to why the AfD has so far failed to use the crisis for its own benefit. First, the voters of the AfD are internally divided on their policy preferences. Second, voter–party incongruence makes previous supporters withdraw their support from the AfD and vote in accordance with their preferences. Third, the likelihood of withdrawing support from the AfD is higher in regions especially affected by the pandemic, leading to a higher vote share decline in these regions.

## Descriptive Evidence

In order to test the first and second hypotheses, we use a novel and comprehensive weekly survey data on voter attitudes toward government measures to combat the COVID-19 pandemic in 2020 and 2021. The third hypothesis is tested with data on regional infection rates and vote share changes of the AfD between 2017 and 2021. Descriptive insights based on these data are provided in the following sections.

### Attitudes to COVID-19 Measures

**Fig. 2:**
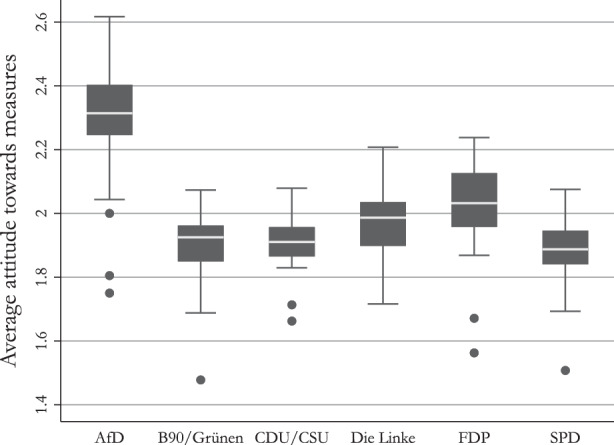
Average attitudes of voters to COVID-19 measures

To assess voter attitudes to COVID-19 measures, we employ the ‘Trendfragen Corona’ survey by the Press and Information Office of the Federal Government of Germany (Presse- und Informationsamt der Bundesregierung, 2021). The survey was launched in week 12 of 2020 as a weekly survey and was later reduced to a bi and quadweekly survey.^3^ Three questions are especially relevant. First, the question on the assessment of the current measures to contain the virus.^4^ The possible answers are “appropriate”, “go too far”, and “do not go far enough” with the additional answers “don’t know” and “no answer”. We recode the answers to range from 1 “do not go far enough” over 2 “appropriate” to 3 “go too far”. The second question of interest asks respondents who they voted for in the last federal election of 2017. This question is used to identify the voters of the AfD. The third and last question we take from the survey asks respondents to indicate who they are planning to vote for in the next federal election of 2021. We use these questions to identify voters who previously voted for the AfD and now indicate that they will vote for a different party. To provide a first descriptive insight into the data we plot the average voters’ preference by party support in the 2017 federal election. The results are displayed in Fig. 2.

**Fig. 3:**
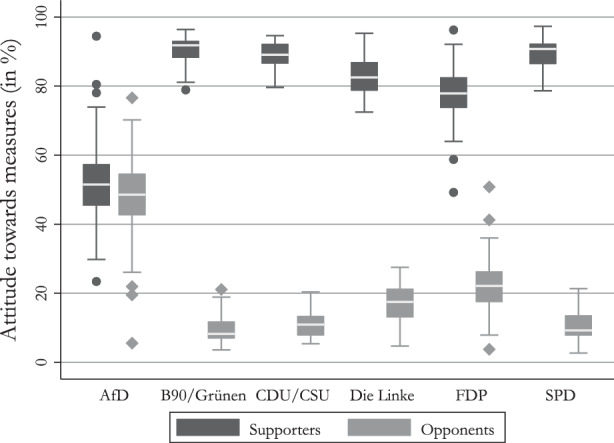
Detailed attitudes of voters to COVID-19 measures

Figure 2 shows that AfD voters – on average – perceive the measures to contain the spread of Corona virus as too far reaching, while the supporters of other parties – on average – show a strong tendency to perceive the measures as appropriate or even not going far enough. This is also the case over the course of the pandemic (see Fig. 10). We further analyze the comparatively large range found on the part of AfD voters by grouping the respondents by their answers. In detail, we group the respective party voters into supporters and opponents of the measures taken against the COVID-19 pandemic. We code respondents who answered that the measures are going to far as opponents of the measures, and respondents who stated that they think the current measures are appropriate or even prefer stronger measures as supporters. Figure 3 plots the percentage of voters by party group.

**Fig. 4:**
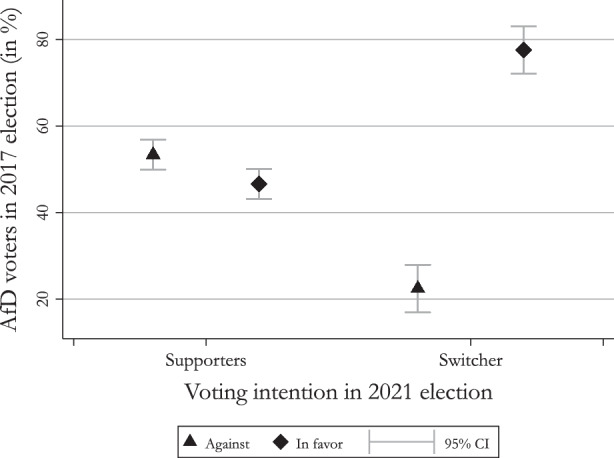
Average AfD supporter and switcher attitude

Figure 3 shows that the voters of the AfD are more divided on the issue than voters of other parties. The only party that shows a slight division in its voter base is the FDP. This finding provides evidence in support of our first hypothesis on the internal division of the AfD’s voter base. The internal division of the AfD becomes even more evident when comparing the voters who supported the AfD in 2017 and still indicate that they will vote for the AfD in the 2021 federal election to the ones that indicated to switch their support to another party. In order to compare these two groups, we subdivided our sample into continuing supporters and vote switchers and plot the percentage of respondents who are in favor of the measures against the ones who are against the measures. The results are shown in Fig. 4.

The figure again strikingly shows the division among the supporters of the AfD with 53% against and 47% in favor of the measures taken by the government. Even more interesting, the respondents who indicated a switch to another party display a clear pattern of favoring the measures by the government. Only around 22% of the switchers state that they are against the measures, while the vast majority of switchers, i.e., 78%, is in favor of the government’s policy response. Not only does this finding further support our first hypothesis on the internal division of the AfD’s voter base, it also sheds some first light on the second hypothesis according to which the support of measures is correlated with withdrawing support from the AfD. Additionally, it draws attention to the fact that a considerable amount of AfD voters is against the COVID-19 measures. Even more importantly, the group of stable supporters also consists of voters that are in favor of the government’s measures. Based on the assumption that voters select parties based on issue proximity, this can only be explained by the fact that these voters seemingly place a greater importance on the AfD’s policy position towards the party’s core issues of migration and nationalism despite these issues being less salient in the COVID-19 pandemic.

### Vote Share Change

To test our second hypothesis, we utilize data on the federal election vote share of the AfD on the regional level. For 2017, we retrieve the county-level AfD vote share from the Federal Election Commissioner’s Office. For 2021, the data has yet to be released from this source, so we based our analysis on the pooled county-level data compiled by ZEIT Online and Fusionbase.^5^ Using both data sources, we compile a dataset, which contains the AfD federal election results for 2017 and 2021 in the 401 German counties.

To compare the performance of the AfD, we calculate the vote share difference between 2021 and 2017 for each county. Motivated by various contributions that underscore the differences in the electoral support between eastern and western Germany (Betz and Habersack 2019; Pesthy et al. 2021; Weisskircher 2020) and our previous considerations on differences between East and West, we aggregate the regional results by eastern and western Germany separately and in total. In doing so, we find that the AfD, on average, lost 2.12 percentage points of votes in western German counties, while the AfD could, on average, increase its vote share by about 2.18 in eastern German counties. In total, the AfD lost 1.31 percentage points from 2017 to 2021 (see also Fig. 11). This vote loss is carried by the electoral losses in western Germany, which cannot be compensated by the electoral gains in eastern German counties. This resonates with the findings of other contributions and is in line with our argument that right-wing attitudes, as well as political disenchantment and distrust, still matter and are arguably different between eastern and western Germany.

From this finding, we can already conclude that the stagnating and sometimes declining support for the AfD over the course of the pandemic has not been a uniform trend across Germany but is largely constrained to western Germany, while the opposite is true for eastern Germany. We account for this by later also splitting the sample into eastern and western German counties to calculate the correlation between pandemic severity and AfD vote share difference individually.

### Infection Rates

**Fig. 5:**
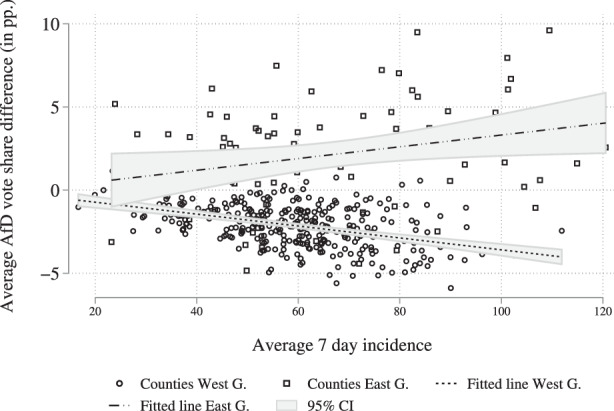
Correlation between average incidence and vote share difference

Testing the third hypothesis requires us to define the regional severity of the pandemic. The severity of the pandemic can be defined in multiple ways. Focusing on Germany, we reason that the spread of the virus and the severity of the pandemic can be accurately described by the infection rate. The data on infection rates comes from the German Federal Statistics Office.^6^ In detail, we use the 7‑day incidence variable to determine the severity of the pandemic on the county level. We calculate the average 7‑day incidence from the 1st of March 2020 until the federal election on the 26th of September 2021. The data is missing for one county. On average, western German counties had a 7-day incidence of 59.96, while eastern German counties witnessed an average 7‑day incidence of 68.77 (see also Fig. 12). With a view to the election results, we can conclude that the increased vote share of the AfD in western and eastern Germany correlates with the severity of the pandemic, with eastern Germany witnessing positive vote share differences and higher 7‑day incidence rates, while western Germany displays a negative vote share difference and a lower 7‑day incidence rates. What is of special interest at this point is whether this correlation also holds on the county level. To shed some light on this consideration, Fig. 5 plots the county average 7‑day incidence against the difference in the AfD’s electoral performance.

Figure 5 strikingly shows that the correlation runs in different directions. While the average 7‑day incidence rate is negatively correlated with AfD vote share differences, i.e., correlated with losses, the average 7‑day incidence is positively correlated with AfD vote share difference in eastern Germany. In conclusion, this means that while high infection rates in western German are correlated with a significant drop in AfD support, high infection rates in eastern Germany are correlated with a significant increase in AfD voting. While the previous sections suggest systematic differences in right-wing populist voting between eastern and western Germany, we nonetheless expected a similar – although eventually weaker – relationship between pandemic severity and AfD vote share decline. The reverse relationship uncovered in Fig. 5 is, therefore, somewhat surprising and will be further addressed in our econometric models.

Additionally, the negative correlation found in western Germany is an important finding, as this speaks against a grave case of reverse causality. Reverse causality is, of course, a concern, as people who reject government measures and do not comply with them will increase the infection rates in the respective regions and might also turn to supporting the AfD over the course of the pandemic. While this mechanism can nonetheless be at work in eastern Germany, it cannot explain that regions with higher infection rates – especially in western Germany – see lower support for the AfD in 2021 than in 2017. Irrespective of this, it is possible that the positive correlation in eastern Germany is – at least in part – based on reverse causality with voters rejecting and not complying with government regulations, and thereby contributing to the spread of the virus, being drawn towards the policy position of the AfD.

## Estimation

Moving to the econometric models, we test our second hypothesis by calculating the probability that a voter who voted for the AfD in the 2017 election supports a party other than the AfD when asked about his or her voting intention in the 2021 election. We regress this vote switching variable (*Switching*) on the individual voter’s attitude towards the COVID-19 measures by the government (*Attitude*) using a logistic regression. We account for the panel-like structure in our data by including week-fixed effects. As we assume our sample to be rather heterogeneous, we employ robust standard errors. We also control for possible confounding variables by including a respondent’s gender (*Male*), age (*Age*), employment type (*Worker*), education (*Low education*), and income (*Low income*). We also control for the differences between eastern and western Germany through region fixed effects. The baseline model is defined by:

$$\begin{aligned}\displaystyle\textit{Switching}_{i,r,w}=\alpha+\beta_{1}\textit{Attitude}_{i,r,w}+\beta_{2}\chi^{j}_{i,r,w}+\omega_{r}\gamma_{r}+\pi_{w}\lambda_{w}+\varepsilon,\end{aligned}$$

with the logit function given with

$$g(\textit{Switching}_{m})=\log\left(\frac{\textit{Switching}_{m}}{1-\textit{Switching}_{m}}\right)\quad m=1,{\ldots},N,$$

where *Switching* is the vote switching away from the AfD of an individual $i$ in a German region $r$ in a week $w$ that is regressed on the attitude of the individual voter towards the COVID-19 measures (*Attitude*). Additionally, with $\chi$, a vector of control variables $j$ is included. Further, terms denoting region ($\gamma$) and week-fixed effects ($\lambda$) are included with the corresponding coefficients.

The second set of regression models analyzes the correlation between region specific severity of the pandemic and voter migration away from the AfD, as formulated in the third hypothesis. The response variable is the change in the AfD’s vote share (*VoteShare*) on the regional level between 2017 and 2021. The change in vote share is regressed on the average 7‑day incidence in the respective county (*Incidence*). Since both variables follow a normal distribution, we employ OLS estimators. We again use robust standard errors and state fixed effects. Motivated by the difference found between eastern and western Germany, we interact the incidence variable with an eastern Germany dummy variable. Apart from this, we also run separate regressions for eastern and western German counties. The baseline model is defined by:

$$\begin{aligned}\displaystyle\Delta\textit{VoteShare}_{r,s}^{f,w,e}=\alpha+\beta_{1}\textit{Incidence}_{r,s}^{f,w,e}+\beta_{2}\textit{East}_{r,s}^{f,w,e}+\beta_{3}\textit{Incidence}*\textit{East}_{r,s}^{f,w,e}+\omega_{s}\gamma_{s}+\varepsilon,\end{aligned}$$

where $\Delta\textit{VoteShare}$ is the difference in the AfD vote share in a given German region ($r$) of a state ($s$) in the full ($f$), western ($w$), or eastern ($e$) German sample. The explanatory variables are the county-specific incidence ($\textit{Incidence}$) and the eastern Germany dummy ($\textit{East}$), as well as the interaction term between both variables ($\textit{Incidence}*\textit{East}$). The dummy variable and the interaction term are only employed in the full sample. Lastly, state fixed effects ($\gamma$) are included with the corresponding coefficient.

The third set of regression models takes this approach one step further and applies a difference-in-differences (DiD) identification strategy (Abadie 2005). For the DiD approach, we identify treated and nontreated regions in our sample. Since our theoretical argument is concerned with particular affectedness, our approach relies on identifying regions that were especially hit by the pandemic. For our main analysis, the cutoff is an average 7‑day incidence above 72, which is the upper 25th percentile. Since we want to avoid any endogeneity that might occur in defining severity based on public perception, we determine the relative severity of the pandemic with Gaussian measures of dispersion that mark observations at specific points of the distribution. We chose the upper 25th percentile since this bound describes values at the upper end of the distribution, i.e., relatively strong affected regions, while simultaneously including enough observations to run our model.

In the robustness checks, we nonetheless use additional cutoffs at the lower 25th percentile (49), the mean (61), and the mean plus one standard deviation (80). We calculate the DiD estimator by running an OLS regression with a treatment and a time dummy variable. The time variable is 1 if the year is 2021 and 0 if the year is 2017. The treatment variable is 1 if a county’s average 7‑day incidence is above 72. The treatment effect is determined by the interaction between the treatment and time dummy variable. For the difference between eastern and western Germany, we include an additional eastern Germany dummy and add an interaction term with this dummy. The baseline model is defined by:

$$\begin{aligned}\displaystyle\textit{VoteShare}_{r,s}^{f,w,e}=\alpha+\beta_{1}\textit{Treated}_{r,s}^{f,w,e}+\beta_{2}\textit{Time}_{r,s}^{f,w,e}+\beta_{3}\textit{East}_{r,s}^{f}+\beta_{3}\textit{Treated}*\textit{Time}_{r,s}^{f,w,e}+\beta_{3}\textit{Treated}*\textit{Time}*\textit{East}_{r,s}^{f}+\omega_{s}\gamma_{s}+\varepsilon,\end{aligned}$$

where *VoteShare* is the AfD vote share in a given region ($r$) of a state ($s$) in the full ($f$), western ($w$), or eastern ($e$) German sample. The explanatory variables are the treatment (*Treated*) and time (*Time*) dummy, as well as their interaction term ($\textit{Treated}*\textit{Time}$), which gives the average treatment effect. Additionally, we also include with *East* a dummy variable for eastern German counties, which we also include in the interaction term. Lastly, state fixed effects ($\gamma$) are included with the corresponding coefficient.

### Results

The results of the set of regression models concerned with the correlation between attitudes towards the measures and vote switching (Hypothesis 2) are displayed in Table 1. The first model only reports the coefficient of the bivariate regression with AfD vote switching as the response and the attitude towards the measures as the explanatory variable. The coefficient is negative and statistically significant, indicating a negative correlation. From this follows that a higher value on the attitude variable, i.e., rejection of the measures, is correlated with a reduced probability of vote switching, i.e., continuing support for the AfD.

**Table 1:** Logistic regression on AfD vote switching

	Dependent variable: AfD vote switching						
	(1)	(2)	(3)	(4)	(5)	(6)	(7)
Attitude	$-0.609$***	$-0.634$***	$-0.634$***	$-0.601$***	$-0.562$***	$-0.556$***	$-0.534$***
	(0.09)	(0.10)	(0.10)	(0.10)	(0.10)	(0.10)	(0.11)
Male			$-0.079$	$-0.090$	$-0.032$	$-0.103$	$-0.268$
			(0.18)	(0.18)	(0.19)	(0.19)	(0.21)
Age				0.252**	0.218**	0.257**	0.307***
				(0.10)	(0.11)	(0.11)	(0.12)
Worker					$-0.867$**	$-1.006$**	$-0.954$**
					(0.43)	(0.45)	(0.46)
Low education						$-0.093$	$-0.033$
						(0.22)	(0.24)
Low income							$-0.147$
							(0.28)
Observations	1,012	1,012	1,012	1,012	940	922	838
Pseudo R‑squared	0.035	0.070	0.070	0.077	0.082	0.089	0.095
Week-fixed effects	No	Yes	Yes	Yes	Yes	Yes	Yes
Region fixed effects	No	Yes	Yes	Yes	Yes	Yes	Yes

Robust standard errors in parentheses *** $p<0.01$, ** $p<0.05$, * $p<0.1$

The next model introduces week and region fixed effects. This does not change the statistical significance of the coefficient and increases its size only slightly. The following models gradually introduce the control variables, while keeping the week and region fixed effects. While the dummy variable concerned with male respondents has no effect on the size and significance of the coefficient of the attitude variable, the inclusion of the age variable is accompanied by a slight drop in the size of the coefficient. In the next model, the size of the coefficient is further reduced by including the dummy variable that controls for employment status. Both control variables are significant, indicating that a higher age increases the probability of vote switching, while being employed as a worker decreases the probability. The low education and income control variables included in the last two models are not statistically significant and only slightly decrease the size of the coefficient. In sum, the coefficient of the main variable of interest is negative and statistically significant throughout the different model specifications. The size of the coefficient is also fairly stable. From this, we can conclude that supporting the COVID-19 measures taken by the government is correlated with an increased probability of vote switching away from the AfD. This finding lends credible support for our second hypothesis.

**Fig. 6:**
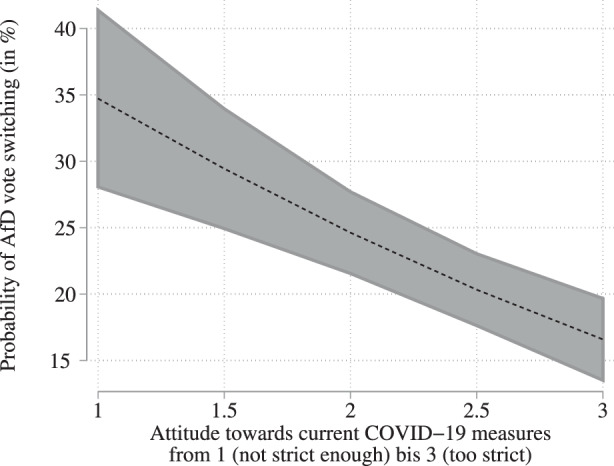
Logistic regression marginal effects

In order to analyze whether this correlation is not only statistically significant but also substantially relevant, we estimate the predicted probability of an AfD voter in the 2017 election withdrawing support in the 2021 election, depending on the attitude towards the COVID-19 measures. While a respondent who voted for the AfD in 2017 and who indicated that the measures are too far reaching has only a predicted vote switching probability of 16.6%, the same respondent has *ceteris paribus* a 34.4% probability of vote switching if he or she feels that the measures should be more far reaching. This predicted probability is visualized in Fig. 6, which shows how the probability of vote switching decreases with increasing rejection of the containment measures. We conclude from this that our second hypothesis does not only find support in terms of statistical significance but also substantial relevance.

Moving from the second to the third hypothesis, we now analyze the correlation between the severity of the pandemic and AfD voting on the county level. The results are displayed in Table 2. The first set of regression models addresses this correlation with a simple OLS regression, where the dependent variable is the change in the AfD’s vote share, and the explanatory variable is the average 7‑day incidence. The first model reports the results for the simple bivariate regression in the full sample without fixed effects. The coefficient is small and positive but not statistically significant. While including state fixed effects increases the size of the coefficient, it nonetheless remains statistically insignificant.

**Table 2:** OLS regression on incidence and AfD voting

	Dependent variable: change in AfD vote share						
	(1) Full	(2) Full	(3) West	(4) East	(5) West	(6) East	(7) Full
Incidence	0.006	0.011	$-0.036$***	0.036**	$-0.020$***	0.084***	$-0.036$***
	(0.01)	(0.01)	(0.00)	(0.02)	(0.01)	(0.02)	(0.00)
Eastern Germany							$-0.268$
							(1.11)
Incidence * Eastern Germany							0.072***
							(0.02)
Observations	400	400	325	75	325	75	400
R‑squared	0.002	0.605	0.200	0.065	0.390	0.389	0.516
State fixed effects	No	Yes	No	No	Yes	Yes	No

Robust standard errors in parentheses *** $p<0.01$, ** $p<0.05$, * $p<0.1$

Surprisingly, the coefficient becomes statistically significant in the following models when employing a sample split between western and eastern Germany. Even more surprising, and replicating the previous descriptive findings, the correlation is negative in the western German sample and positive in the eastern German sample. This means that regions with high average 7‑day incidences witnessed a decline in AfD voting in western Germany, while the opposite is true for eastern Germany. The next two models introduce state fixed effects. Including the fixed effects slightly decreases the size of the coefficient in the western German sample and more than doubles it in the eastern German sample. The statistical significance is not impacted negatively by including the fixed effects. Based on these findings, the last model is run in the full sample but with an additional interaction term between an eastern Germany dummy and the incidence variable. These results replicate the previous finding from the sample split, with a negative correlation in case of the eastern Germany dummy being 0, i.e., the western German sample, and a positive correlation in case of the eastern German dummy being 1, i.e., the eastern German sample. This finding partially supports the third hypothesis, as we find a negative impact of the COVID-19 incidences on the AfD vote share, but this effect is exclusively restricted to western Germany.

**Fig. 7:**
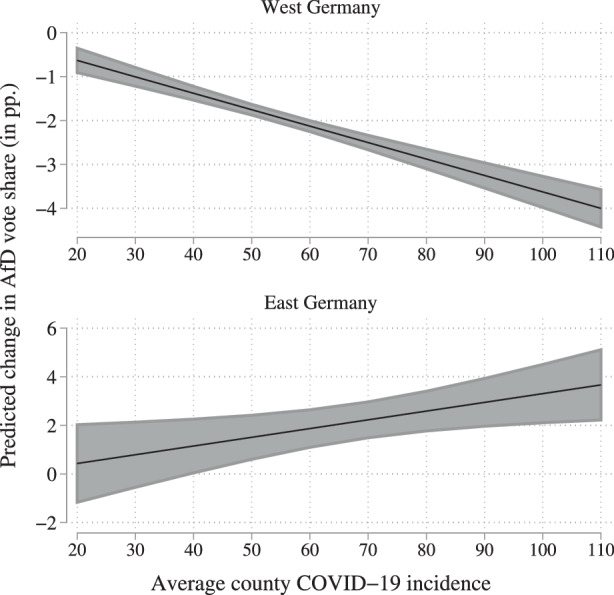
OLS regression marginal effects

After having established the statistical significance of the correlation between negative vote share differences and high average incidences in western Germany and a positive vote share difference and high average incidence in eastern Germany, we again want to analyze the substantial relevance of the correlation found. In order to do so, we calculate the predicted change in the AfD’s vote share in eastern and western German counties in the case of a one-standard deviation increase (18.4) of the average 7‑day incidence. In western Germany, a one-standard deviation increase is correlated with a 0.5-standard deviation decrease (0.69 pp.) in the AfD vote share difference. In eastern Germany, a one-standard deviation increase correlates with a 0.2-standard deviation increase (0.67 pp.). The substantial relevance of this correlation is further illustrated in Fig. 7. This finding strongly supports the hypothesized correlation in terms of statistical significance and substantial relevance for western Germany and reveals an inverse pattern in eastern Germany.

In the last step, we employ a DiD approach to further analyze the correlation between the average 7‑day incidence and changes in the AfD’s vote share. The response variable is the AfD’s county-level vote share. The explanatory variable of interest is the interaction term between treatment and time dummy. The results of the regression analysis are displayed in Table 3. The first model reports the simple DiD regression. The interaction term, indicating the treatment effect, is positive but not statistically significant. This does also not change when including the state fixed effects in the next model.

**Table 3:** Diff-in-diff regression on incidence and AfD voting

	Dependent variable: AfD vote share						
	Treatment cut-off at incidence 72						
	(1) Full	(2) Full	(3) West	(4) East	(5) West	(6) East	(7) Full
Treated	5.538***	2.593***	2.684***	6.932***	2.424***	6.383***	2.661***
	(0.70)	(0.36)	(0.37)	(0.78)	(0.34)	(1.05)	(0.37)
Time	$-1.432$***	$-1.432$***	$-1.906$***	1.435	$-1.906$***	1.435	$-1.906$***
	(0.36)	(0.21)	(0.21)	(0.87)	(0.18)	(0.90)	(0.21)
Treated*Time	0.436	0.436	$-1.011$**	1.700	$-1.011$**	1.700	$-1.011$**
	(1.17)	(0.53)	(0.49)	(1.34)	(0.45)	(1.35)	(0.49)
East							8.156***
							(0.45)
Time*East							3.341***
							(0.89)
Treated*East							4.246***
							(0.85)
Treated*Time*East							2.711*
							(1.42)
Observations	800	800	650	150	650	150	800
R‑squared	0.605	0.200	0.0655	0.390	0.389	0.516	0.793
State fixed effects	No	Yes	No	No	Yes	Yes	No

Robust standard errors in parentheses *** $p<0.01$, ** $p<0.05$, * $p<0.1$

The following models again employ a sample split with and without state fixed effects. Other than in the previous simple OLS regression, the DiD approach does not report a positive and statistically significant correlation of the treatment in eastern Germany. Contrary to this, the coefficient is again negative and statistically significant in western Germany. In the last model, we turn to the full sample with an eastern German dummy variable interaction term. For western Germany, the interaction term between time and treatment remains negative and statistically significant, replicating the previous finding. In contrast, the interaction term for eastern Germany has a positive coefficient that is statistically significant only at the 0.1 level. From this follows that the treatment of experiencing a particularly high incidence is correlated with a decrease in the AfD’s vote share in western Germany but not in eastern Germany. If anything, the treatment of a high average incidence is correlated with an increase in the AfD’s vote share in eastern Germany.

**Fig. 8:**
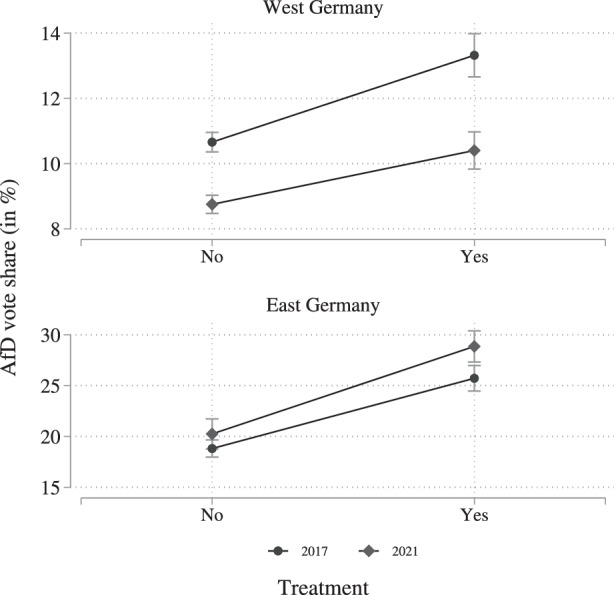
Diff-in-diff regression marginal effects

**Fig. 10:**
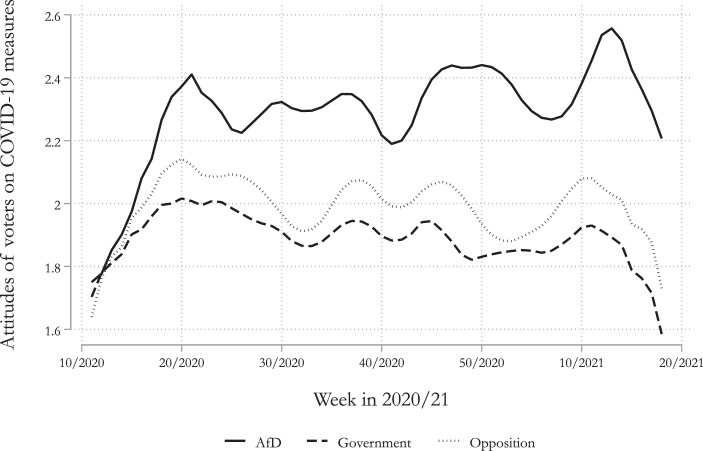
Attitudes to COVID-19 measures over time. *Notes:* The average values are plotted by calculating a moving average using two lagged and forwarded terms and the current observation in the filter

**Fig. 11:**
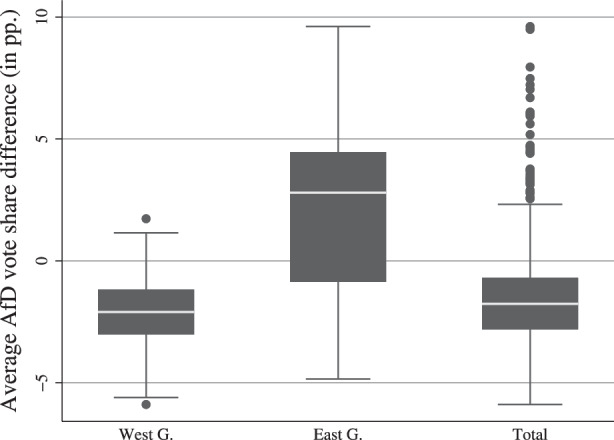
Change in AfD vote share from 2017 to 2021

**Fig. 12:**
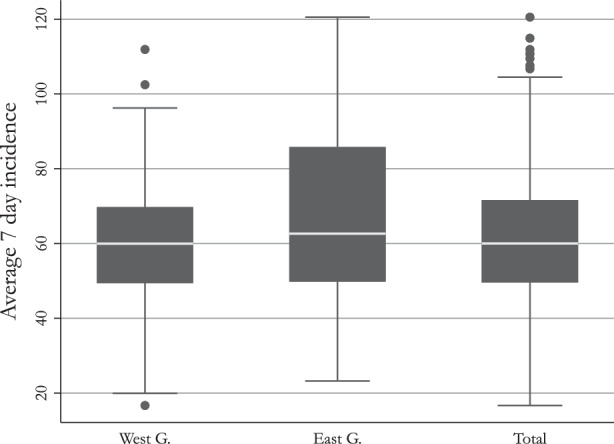
Average incidence between onset of the pandemic and federal election

After having established the robust statistical significance of the correlation in western Germany and the less robust correlation in eastern Germany, we again turn towards calculating the marginal effects. The marginal effects for the DiD regression are displayed in Fig. 8. First focusing on western Germany, the figure shows that the AfD vote share in 2021 (gray diamonds) was lower in treatment and nontreatment counties, when compared to 2017 (black dots). The particular strength of the DiD approach is to calculate whether the difference within these differences is statistically significant while controlling for county specific differences. The figure shows that the difference between the 2017 and 2021 average vote share is considerably larger in treated counties (right-hand side), when compared to the difference in the not treated counties (left-hand side).

Focusing on the eastern German counties, we find that the AfD vote share in 2021 (gray diamonds) is higher than the vote share in 2017 (black dots). Other than in western Germany, the difference is larger in the treated counties (right-hand side) than in the not treated counties (left-hand side), although this positive difference is not statistically significant at the .05 level. From the regression analysis, we also know that the difference between the observations is only statistically significant at the 0.1 level. In terms of substantial relevance, we can conclude that experiencing a particularly high incidence is correlated with a reduction in the AfD’s vote share by about 3 percentage points in western Germany. In eastern Germany, however, being particularly affected by the pandemic, it is, if anything, correlated with an increase in the AfD’s vote share also by about 3 percentage points.

In conclusion, our empirical analysis finds considerable support for the above derived hypotheses with one exception. We found that the AfD consistently took a position outside the political consensus of other parties throughout the course of the pandemic and that the voters of the AfD show a distinct internal division regarding their attitudes towards the measures taken against the COVID-19 pandemic. The logistic regression analysis also supported the hypothesis that being in favor of the government measures significantly increases the likelihood of vote switching away from the AfD. The hypothesized correlation between particularly high incidences and the loss of votes of the AfD, however, could only be found in western Germany. Contrary to this, in eastern Germany, the correlation is reversed and considerably weaker in statistical terms, leading us to partially reject the last hypothesis.

### Robustness of Results

In the robustness section of our analysis, we focus on the previous DiD results and establish different treatment cutoffs. In detail, we run three additional regression analyses. The treatment cutoff points for these regressions are 49 (lower 25th percentile), 61 (mean), and 80 (mean plus one standard deviation). The regression with the cutoff of 49 is displayed in Table 4. The models show a similar size and statistical significance of the time and treatment variable interaction term for the western German subsamples and the full sample with the eastern German dummy variable. Other than in the previous models, the subsamples and interaction terms addressing the correlation in eastern Germany are not even statistically significant at the 0.1 level. This finding supports our previous results on the negative correlation between the pandemic shocks and AfD voting found for western Germany. The findings for eastern Germany, however, increase our skepticism about the statistical significance of positive correlation in eastern Germany.

Moving to the DiD regression with the cutoff of 80 displayed in Table 5, we find fairly similar results as in our main analysis. While the results for Germany remain stable, the results for eastern Germany become statistically significant again on the 0.1 level. This only changes in the third robustness check for the DiD regression displayed in Table 6. In this robustness check, the eastern German subsample regressions report a positive and significant coefficient on the 0.1 level and even on the 0.05 level. Simultaneously, the interaction term coefficient in the western German subsample drops to the 0.1 level. This can be explained with the reduced number of western German counties with an average incidence above 80, as only 38 western German counties reported an average incidence above 80.

**Fig. 13:**
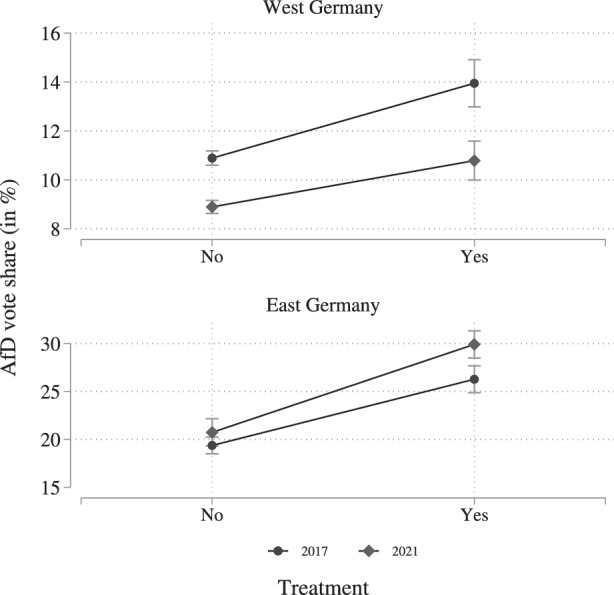
Robustness: Diff-in-diff regression marginal effects

Additionally, we again plot the marginal effects for the DiD robustness check regressions that use the cutoff of 80. The results are plotted in Fig. 13 and are consistent with our previous findings. In the model concerned with western Germany, we find a difference within the treatment group of around 3 percentage points. In the case of eastern Germany, with 4 percentage points, the effect size is even slightly larger than in the main analysis. However, especially the last finding has to be taken with a grain of salt as the statistical significance of the correlation is not robust against different model specifications.

Lastly, it is important to address other long-term developments that could potentially affect the vote share of the AfD. The AfD was also not able to substantially gain votes in the state legislative elections prior to the federal election of 2021. Our proposed theoretical model and empirical analysis does not propose that the AfD’s stagnating election results can be entirely attributed to its performance in the COVID-19 pandemic. Rather, we provide theoretical arguments as to why voters might (additionally) withdraw their support from the AfD due to incongruences between the demand and supply side. Methodologically, this creates an identification problem, as it is initially not clear whether the loss of votes is related to a long-term decline of populist support or are at least partially rooted in the pandemic shock and the populist response to it. We address this issue in two ways. First, we provide evidence on the individual level that people are more likely to turn away from the AfD with increasing support of the COVID-19 measures introduced by the government. At the same time, it is unlikely that people supporting the government measures are systematically more aware and dismissive of the AfD’s scandals, internal division, or public perception. Second, the same is true on the regional level, as a bias in the DiD regression could only arise, if the AfD’s lack of support would be positively correlated with higher infection rates.

In sum, the robustness checks concerned with the DiD regression further strengthen our confidence in our previous findings. While we find strong and robust support for a negative correlation between pandemic severity and AfD support in western Germany, we cannot reach the same conclusion for eastern Germany. Although the correlation found for eastern Germany is substantial, the positive correlation has to be treated carefully due to the lack of robust statistical significance.

## Conclusion

In this paper, we asked why the COVID-19 crisis does not have a positive impact on AfD support. Our main argument in answering this question is that the AfD lost voters because its support base is internally divided on the position towards the government’s COVID-19 measures. In detail, we argued that other than GAL-TAN issues like migration, EU integration, or nationalism, around which the AfD has built its support base, the issue of COVID-19 poses a cross-cutting issue that does not easily align with established dimensions of electoral competition. Based on this, we argued that although some voters of the AfD will keep supporting the party based on their rejection of the COVID-19 measures, preference for other positions supplied by the AfD or general detachment from the political system, others will switch their support and vote in accordance with their COVID-19 policy preference. While we reason that these voters will have a higher likelihood of vote switching, we also argued that this trend should be especially observable in regions hit particularly hard by the pandemic. From this, we derived three hypotheses. First, the voter base of the AfD is internally divided on how to react to the pandemic. Second, voters with preferences diverging from the AfD’s policy position on COVID-19 have a higher likelihood of vote switching. Third, the more strongly a region is hit by the pandemic, the higher will be the AfD vote loss in that region.

We tested the hypotheses with novel and comprehensive survey data on voter attitudes, infection rates, and voting behavior and intention on a weekly basis in 2020 and 2021. In detail, we used micro-level survey data on attitudes toward COVID-19, previous vote choice, and future voting intentions. Additionally, we included county-level data on the federal election results of 2017 and 2021, as well as data on the county-level 7‑day COVID-19 incidence rate.

The first hypothesis was evaluated with a descriptive analysis of voter attitudes. Our results suggest that the AfD’s support base shows a strong and unparalleled internal division on the preferences towards COVID-19 policy measures. Taking this finding one step further, running logistic regression analysis supported our proposition that voters with policy preferences diverging from the AfD’s position were significantly more likely to withdraw their support from the AfD. Finally, we used OLS regression analyses and a DiD approach to examine whether the severity of the pandemic had an impact on the AfD’s election results in the 2021 election. As discussed, we were able to show that this is the case for western Germany. However, we did not find a robust, statistically significant correlation in eastern Germany. Thus, our third hypothesis has be partially rejected.

Despite employing robustness checks and implementing a fairly robust identification strategy, our analysis has some weaknesses and blind spots, which increases the need for further research of the relationship between the loss of votes for the AfD and the COVID-19 pandemic. An important issue that we have to address is reverse causality. Recent research has found that AfD strongholds in eastern and western Germany in the 2017 election are also the counties that have been hit especially hard by the pandemic (Richter et al. 2021). This is in line with our finding, as we can show that a certain number of voters in these affected regions will withdraw their support, while a considerable number will continue to support the AfD and also probably not strictly comply with public health regulations (see also Gollwitzer et al. 2020) or have a lower risk perception due to partisan biases (Barrios and Hochberg 2020).

However, while we can arguably rule out the possibility of reverse causality in western Germany due to the direction of the hypothesized and found correlation, this is not the case for eastern Germany. In eastern Germany, reverse causality could be an issue but, of course, only against the backdrop of the nevertheless not statistically robust correlation. Based on the DiD regression, this would still mean that additional voters switched to the AfD in the wake of rising 7‑day incidence rates, which were, in turn, also carried by AfD voters. Hence, reverse causality is not as big of an issue for our analysis as it might seem at first glance. Nonetheless, upcoming state elections should be utilized in future research for a more robust empirical identification strategy that also controls for reverse causality, especially in eastern Germany.

Regarding the generalizability of our results, we can only make suggestions on how the mechanism might work in other countries. This goes especially for populists in government, who have seemingly mishandled the pandemic but are to a large extent still in office (Bayerlein et al. 2021). Therefore, our findings are only transferable to a limited extent. Future contributions should, therefore, analyze how the pandemic affects populist parties in other countries and not only in opposition but also in government. However, with regard to the transferability of the proposed mechanism, we already find similar tendencies of an internal division with a view to the AfDs position on the Russian invasion of Ukraine in early 2022 and President Putin. It may, therefore, be fruitful in future research to apply the proposed mechanism to other exogenous shocks outside the core ideology of populist parties.

Lastly, the uncovered difference between eastern and western Germany demand more research on why the AfD has not lost support in eastern German counties. This question further underscores that different mechanisms of political support are at work when comparing eastern Germany to western Germany. This is, of course, in line with existing literature that already provides diverging findings for eastern Germany and western Germany concerning political attitudes and AfD support (see, e.g., Betz and Habersack 2019; Pesthy et al. 2021). With a view to the COVID-19 pandemic, we uncovered another mechanism that is not transferable from one German region to the other. One possibility for this nontransferability could lie in differences in the AfD’s support structures, with voters possibly ignoring potential policy inconsistencies as long as their demand for nativist, antimigrant attitudes, and political dissatisfaction is satisfied, while western German AfD voters are more policy-oriented and change their vote intentions based on issue salience. Either way, further research is needed to analyze the uncovered differences.

Irrespective of the remaining questions, we can conclude that a considerable number of AfD supporters jumped ship in the wake of the COVID-19 pandemic. This finding has important implications for the impact of exogenous shocks on electoral competition and also on the future of populist parties. In our paper, we have provided evidence that exogenous shocks like the COVID-19 pandemic have the potential to shake up the party system, as they are usually cross-cutting issues that increase the likelihood of voter realignment. Additionally, we were also able to show that populist party support is not based on blind allegiance, but is, at least for some voters, rooted in their policy preferences. If these preferences are not in line with the populist party’s position and also become salient, voters seemingly do not hesitate to defect from populist parties. The downside of this is that it took an existential crisis for some voters to realize that sailing on with a populist party could not lead to the promised land but maybe to certain doom.

## Figures and Tables

**Fig. 9:**
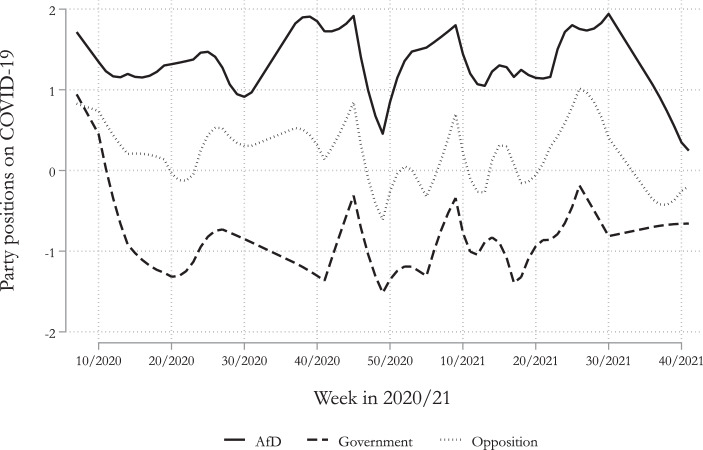
Party positions on COVID-19 measures over time. *Notes:* The average values are plotted by calculating a moving average using two lagged and forwarded terms and the current observation in the filter

## Appendix

### Party Positions on COVID-19 Measures

To determine the party positions on COVID-19 measures, we analyze plenary protocols that contain every speech held in the German federal parliament between the 1st of March 2020 and the 7th of September 2021.^7^ In total, this gives us the speeches from 93 regular plenary sessions over the course of the pandemic. The policy positions are determined with the *Wordfish* tool introduced by Slapin and Proksch (2008). This tool is by now commonly used to determine the overall policy position of parties from manifestos and speeches on a range of subjects but also single issues (see e.g. Debus and Tosun 2021). Since we are interested in the policy positions towards the measures taken against the COVID-19 pandemic, we only included parliamentary speeches that contain one of the following words: COVID-19, COVID, Corona, Pandemie, Virus, SARS-CoV, SARS. We validated this identification strategy by a random sample review.

To determine the positions in a given week, we grouped the speeches by week and party. Further, we include a grouping variable that links different speeches to the parties. As a result, this gives us the relative difference or similarity of texts by party and week, while accounting for the fact that the text of a party in a given week (t) is not independent from the text of the same party in the previous week (t-1). The assumption behind the *Wordfish* method is that the words used in the texts are constant across the time frame of the analysis. We have no reasonable doubt that this is not the case for any word central to the analysis. An additional assumption is that a party ($i$) mentions a word ($j$) in week ($t$) based on a Poisson distribution. This means that the probability of a word being used in a given week does not affect the probability of this word being used again in a following week. Further, this also implies that the same word will be used with a certain probability within a given time frame. Based on these assumptions, the following formula is used to calculate the difference/similarity between texts:

1 $$\begin{aligned}\begin{array}[]{l}y_{j,i,t}\sim\textit{Poisson}(\lambda_{j,i,t})\quad\text{and}\quad\\ \lambda_{i,j,t}=\exp(\alpha_{i,t}+\psi_{j}+\beta_{j}*\theta_{i,t}),\end{array}\end{aligned}$$

where $\lambda_{j,i,t}$ is the frequency of word $j$ in a given text of party $i$ in week $t$. With $\alpha$, a set of party and week-fixed effects is included. Similarly, $\psi$ includes word fixed effects. Further, word specific weights are included with $\beta$, to account for the relative importance ascribed to words by different parties. Lastly, $\theta$ gives the estimated position of a party $i$ at week $t$.

Based on the $\theta$ calculated by *Wordfish*, we can determine the position of a party in a given week. However, it is important to note that *Wordfish* only gives us the difference between texts. Therefore, we need additional knowledge on the positions of parties to determine the difference in their policy positions. For example, using *Wordfish* we might find that the texts of two parties on redistribution are different to a certain extent. Based on this, we do not know whether party A, with a higher $\theta$ score, is more in favor of redistribution than party B. We only know that the texts of both parties differ from one another. Based on prior knowledge, we might know that party A favors liberal economic policies, while party B is in favor of welfare state measures. Now we can use the calculated $\theta$ to assume that a high $\theta$ indicates more liberal economic policies while a low $\theta$ is an indicator of more redistribution. If we now include a third party C, we can determine whether this party is closer to A or B when it comes to redistribution.

The average $\theta$s of our analysis are displayed in Fig. 1. As expected, the figure shows a considerable variance between party positions. Just as in the example, we have to determine what this difference means. In order to do so, we focus on parties whose positions we know. Given that the coalition government during the COVID-19 pandemic consisted of the CDU/CSU and the SPD, and that speeches from politicians of both parties are likely to support the government’s policy measures taken to contain the pandemic, we assume that low $\theta$s indicate agreement with the measures taken against COVID-19. Conversely, high values indicate disagreement with the measures taken.

### Additional Figures and Tables

**Table 4:** Robustness: Diff-in-diff regression on incidence and AfD voting

	Dependent variable: AfD vote share						
	Treatment cut-off at incidence 49						
	(1) Full	(2) Full	(3) West	(4) East	(5) West	(6) East	(7) Full
Treated	3.056***	2.094***	2.537***	5.130***	1.830***	2.945***	2.537***
	(0.51)	(0.37)	(0.28)	(0.80)	(0.37)	(0.91)	(0.28)
Time	$-0.656$	$-0.656$*	$-1.281$***	2.104*	$-1.281$***	2.104*	$-1.281$***
	(0.69)	(0.34)	(0.32)	(1.16)	(0.30)	(1.12)	(0.32)
Treated*Time	$-0.861$	$-0.861$**	$-1.100$***	0.103	$-1.100$***	0.103	$-1.100$***
	(0.85)	(0.42)	(0.39)	(1.56)	(0.37)	(1.47)	(0.40)
East							8.598***
							(0.56)
Time*East							3.385***
							(1.19)
Treated*East							2.592***
							(0.84)
Treated*Time*East							1.202
							(1.60)
Observations	800	800	650	150	650	150	800
R‑squared	0.045	0.769	0.245	0.183	0.394	0.333	0.739
State fixed effects	No	Yes	No	No	Yes	Yes	No

Robust standard errors in parentheses *** $p<0.01$, ** $p<0.05$, * $p<0.1$

**Table 5:** Robustness: Diff-in-diff regression on incidence and AfD voting

	Dependent variable: AfD vote share						
	Treatment cut-off at incidence 61						
	(1) Full	(2) Full	(3) West	(4) East	(5) West	(6) East	(7) Full
Treated	3.521***	2.821***	2.627***	6.662***	2.341***	6.064***	2.627***
	(0.49)	(0.31)	(0.27)	(0.76)	(0.26)	(0.94)	(0.27)
Time	$-1.107$**	$-1.107$***	$-1.646$***	1.332	$-1.646$***	1.332	$-1.646$***
	(0.46)	(0.26)	(0.24)	(0.89)	(0.20)	(0.94)	(0.24)
Treated*Time	$-0.422$	$-0.422$	$-0.966$***	1.637	$-0.966$***	1.637	$-0.966$***
	(0.82)	(0.40)	(0.37)	(1.35)	(0.32)	(1.33)	(0.37)
East							8.459***
							(0.46)
Time*East							2.977***
							(0.91)
Treated*East							4.035***
							(0.80)
Treated*Time*East							2.604*
							(1.39)
Observations	800	800	650	150	650	150	800
R‑squared	0.086	0.793	0.303	0.475	0.458	0.513	0.797
State fixed effects	No	Yes	No	No	Yes	Yes	Yes

Robust standard errors in parentheses *** $p<0.01$, ** $p<0.05$, * $p<0.1$

**Table 6:** Robustness: Diff-in-diff regression on incidence and AfD voting

	Dependent variable: AfD vote share						
	Treatment cut-off at incidence 80						
	(1) Full	(2) Full	(3) West	(4) East	(5) West	(6) East	(7) Full
Treated	7.046***	2.766***	3.057***	6.906***	2.690***	5.757***	3.057***
	(0.89)	(0.45)	(0.51)	(0.85)	(0.46)	(0.96)	(0.51)
Time	$-1.515$***	$-1.515$***	$-1.994$***	1.364	$-1.994$***	1.364	$-1.994$***
	(0.35)	(0.20)	(0.20)	(0.85)	(0.17)	(0.86)	(0.20)
Treated*Time	1.222	1.222*	$-1.168$*	2.275*	$-1.168$*	2.275*	$-1.168$*
	(1.56)	(0.68)	(0.67)	(1.33)	(0.61)	(1.31)	(0.67)
East							8.475***
							(0.47)
Time*East							3.358***
							(0.87)
Treated*East							3.849***
							(0.98)
Treated*Time*East							3.443**
							(1.48)
Observations	800	800	650	150	650	150	800
R‑squared	0.225	0.794	0.231	0.508	0.427	0.529	0.790
State fixed effects	No	Yes	No	No	Yes	Yes	Yes

Robust standard errors in parentheses *** $p<0.01$, ** $p<0.05$, * $p<0.1$

## Footnotes

1 For the necessary distinction between (co)governing populist parties and those in opposition, see Bobba and Hubé (2021).

2 See the Appendix for further details on the application of the *Wordfish* tool and the calculation of the positions.

3 In detail, the surveys were administered in the following weeks of 2020: 12, 13, 14, 15, 16, 17, 18, 19, 20, 22, 24, 26, 28, 32, 37, 41, 45, 47, 51 and the following weeks of 2021: 2, 4, 6, 8, 10, 12, 13.

4 The wording of the question is: “What is your assessment of the current policy measures to contain the Corona virus? In your opinion, are the measures taken appropriate, do they go too far, or do they not go far enough?”.

5 https://www.zeit.de/politik/deutschland/2021-09/ergebnisse-bundestagswahl-gemeinde-karte.

6 https://www.corona-daten-deutschland.de/dataset/infektionen_kreise.
